# Examining the implementation of NICE guidance: cross-sectional survey of the use of NICE interventional procedures guidance by NHS Trusts

**DOI:** 10.1186/s13012-015-0283-4

**Published:** 2015-06-30

**Authors:** Karin Lowson, Michelle Jenks, Alexandra Filby, Louise Carr, Bruce Campbell, John Powell

**Affiliations:** York Health Economics Consortium, Enterprise House, Innovation Way, University of York, Heslington, York YO10 5NQ UK; National Institute for Health and Care Excellence, 10 Spring Gardens, London, SW1A 2BU UK

**Keywords:** Guideline, Implementation, Evidence-based practice, Dissemination, Organisational behaviour

## Abstract

**Background:**

In the UK, NHS hospitals receive large amounts of evidence-based recommendations for care delivery from the National Institute for Health and Care Excellence (NICE) and other organisations. Little is known about how NHS organisations implement such guidance and best practice for doing so. This study was therefore designed to examine the dissemination, decision-making, and monitoring processes for NICE interventional procedures (IP) guidance and to investigate the barriers and enablers to the implementation of such guidance.

**Methods:**

A cross-sectional survey questionnaire was developed and distributed to individuals responsible for managing the processes around NICE guidance in all 181 acute NHS hospitals in England, Scotland, Wales and Northern Ireland. A review of acute NHS hospital policies for implementing NICE guidance was also undertaken using information available in the public domain and from organisations’ websites.

**Results:**

The response rate to the survey was 75 % with 135 completed surveys received. Additionally, policies from 25 % of acute NHS hospitals were identified and analysed. NHS acute hospitals typically had detailed processes in place to implement NICE guidance, although organisations recognised barriers to implementation including organisational process barriers, clinical engagement and poor targeting with a large number of guidance issued. Examples of enablers to, and good practice for, implementation of guidance were found, most notably the value of shared learning experiences between NHS hospitals. Implications for NICE were also identified. These included making improvements to the layout of guidance, signposting on the website and making better use of their shared learning platform.

**Conclusions:**

Most organisations have robust processes in place to deal with implementing guidance. However, resource limitations and the scope of guidance received by organisations create barriers relating to organisational processes, clinician engagement and financing of new procedures. Guidance implementation can be facilitated through encouragement of shared learning by organisations such as NICE and open knowledge transfer between organisations.

**Electronic supplementary material:**

The online version of this article (doi:10.1186/s13012-015-0283-4) contains supplementary material, which is available to authorized users.

## Background

Various organisations exist worldwide that produce evidence-based guidance and recommendations about health care. In the UK, the National Institute for Health and Care Excellence (NICE) provides evidence-based guidance on the most effective ways to prevent, diagnose and treat disease and ill health, reducing inequalities and variation in practice. The NICE interventional procedures (IP) programme produces guidance “designed to protect patients’ safety and support people in the NHS in the process of introducing new procedures” [1]. The guidance makes recommendations about whether interventional procedures used for diagnosis or treatment are safe enough and work well enough for routine use and the circumstances in which they should be used. IP guidance applies to England, Wales, Northern Ireland and Scotland and is not subject to local review before dissemination. NICE makes no recommendations about the implementation of IP guidance, but in England, the guidance is enforceable by the Care Quality Commission (CQC), and the NHS Litigation Authority takes adherence to IP guidance into account in risk assessing NHS hospital trusts [2].

Health care organisations face several potential barriers when implementing evidence-based guidance on aspects of care [3–5]. In 2008, a systematic meta-review of the factors influencing the implementation of clinical guidelines, conducted by Francke et al., found that effective guidance implementation strategies often have multiple components and are more effective than those strategies with only one component. Further, guidelines that are easy to understand, require few resources to implement and can be easily trialled are more likely to be implemented. Both the characteristics of professionals and environmental factors influence the success of implementation. For example, when professionals are familiar with the content of the guidance, support is given from peers and superiors and sufficient resources and time are available, the guidance has a better chance of being successfully implemented. However, the review also concluded that the evidence base in this area is still limited, and well-constructed empirical research looking at various implementation strategies is needed [5].

In 2004, Sheldon et al. [3] assessed the extent of NICE guidance implementation across a random sample of 20 NHS hospital trusts. The authors found that failure to implement guidance emerges from a combination of system and organisational and individual factors. Trusts with high compliance to NICE guidelines had common characteristics, such as, a commitment to a process for implementing guidance, identification of a lead clinician early in the NICE guidance development and involvement of clinicians in the guideline development process [3]. Weng et al. undertook a survey of hospitals to identify the level of evidence-based practice implementation in Taiwan and found that a lack of time was the most commonly reported barrier to implementation. The authors reported that this finding was consistent with findings in other countries. Implementation could be improved through training in evidence-based practice, having a professional faculty and awareness and understanding of the merits of evidence-based practice [4].

Research looking at specific cases of guideline implementation has identified similar factors to those cited by Francke et al., Sheldon et al. and Weng et al. [3–5]. For example, Connolly et al. investigated the implementation of post hospital discharge for critical illness rehabilitation guidance in UK intensive care units. Whilst the guidance had been successful in raising the profile of the importance of rehabilitation following critical illness, there had been little change in clinical services following the publication of the guidance. Barriers to providing the recommended services include lack of funding, scarce resources and lack of priority by clinical management teams [6]. Audits and research investigating specific cases of guidance implementation have identified similar themes. These include a lack of management support leading to poorer implementation [7, 8], complex guidance leading to poor implementation [9] and sufficient resources and staff engagement leading to more successful guidance implementation [10, 11].

Enablers and barriers to guidance implementation can be evaluated against theoretical frameworks. Michie et al. [12] proposed a novel method for characterising behaviour change interventions. The behaviour change wheel (BCW) consists of nine intervention functions that allow the behaviour change to take place and seven categories of policy that enable interventions to occur. The BCW considers how and why people change behaviour in professional practice. Given that the aim of implementing guidance is to change behaviour, using the wheel as a theoretical framework can help to evaluate how and when behaviour change interventions (such as guidance) are successfully introduced. Guidelines are one of the policy categories that enable behaviour change.

Therefore, some evidence exists around the barriers to successful implementation of clinical guidance. However, there is a paucity of evidence describing factors enabling successful implementation of guidance (enablers). Given the limited evidence base around the barriers and enablers in the successful implementation of guidance, a self-administered cross-sectional survey of all acute NHS hospitals in the UK was conducted. The survey aimed to evaluate current awareness and use of NICE Interventional Procedures guidance and to identify barriers and enablers to the successful implementation of the guidance. IP guidance was used as a case study example, aiming to draw both specific and generic conclusions to inform future policy and practice regarding the dissemination and implementation of evidence-based guidance. The results of this research, in particular the barriers and enablers to successful implementation, have implications for the implementation of evidence-based guidance more generally.

## Methods

### Survey development

A draft questionnaire was developed iteratively through a targeted literature review of previous studies in this area [3–11, 13–16] and a process of survey validation. Face validity was established through consultation with key informants familiar with the topic under investigation (two implementation consultants at NICE and four individuals working within the NHS involved in guidance implementation). The survey was piloted in a convenience sample (20 participants) who work at the University of York and who have knowledge of the NHS. The survey was revised following expert comments and pilot participant comments. A final review of the revised draft instrument was carried out by all authors and reviewed by two senior NHS staff working in audit and governance roles. The final survey consisted of 20 questions (8 closed and 12 open questions) (see Additional file 1: Survey). NHS ethics approval was not required as this project fell within the definition of service evaluation [17].

The survey was supplemented and informed by a documentary analysis of NHS hospitals’ policies on implementing NICE guidance (specifically IP guidance where possible) carried out by two members of the research team (MJ and AF). Searches were undertaken initially of websites ending in nhs.uk and then more widely to identify hospital policies. A data extraction table was designed and populated with information from each policy, and themes and characteristics were detected. Double data extraction was carried out by both reviewers (MJ and AF), and any discrepancies were resolved through discussion. This review was undertaken to remove the need for hospitals to provide a copy of their policy, thus, reducing the burden on the respondents.

### Survey distribution

An email distribution list was compiled which included Medical Directors, Clinical Governance Leads and individuals within hospitals who oversee NICE guidance management (NICE managers) at all 181 NHS acute hospitals in the UK. The online survey was distributed and hosted by a third party (Qa Research Ltd) specialising in public sector research. The survey was required to be completed once for each NHS acute hospital. In order to get as many responses as possible, the deadline for the survey was extended several times and multiple follow-up reminders were undertaken by email and telephone. A letter was also sent to Medical Directors of all the organisations from the Chairman of the NICE Interventional Procedures Advisory Committee to encourage response to the survey. In total, the survey was open for 17 weeks between June and September 2013.

### Analysis

The data from the online survey responses were imported into and organised and analysed using a spreadsheet (Microsoft® Excel). Quantitative and qualitative analysis were combined, as for example, advocated by Smith [18]. The quantitative analysis was mainly descriptive (means and frequencies), with a chi-squared test to examine whether there was a difference between types of respondent. Content analysis was used for the 12 open questions with free text responses. One of the research team (KVL) reviewed the survey free text responses, and through multiple readings, she identified a large number of common themes (via words, singly or in groups, or sentences) which were revised and grouped into a smaller number of themes. To ensure the processes were rigorous a review checklist was developed and completed [19]. The checklist was developed as the survey methodology did not concern the majority of elements addressed in many published qualitative research checklists. The review of responses and the identified themes was informed by the research objectives, by information collected in the other strands of the project and by the knowledge and experience of the team, with whom the process and results were shared and discussed on an ongoing basis. The qualitative analysis was thus contextualised by both information collected from other phases of the research project and existing knowledge from other similar projects undertaken by members of the research team [13–15]. In practice the qualitative (free text) responses yielded few data from many cases: the majority of questions required brief responses, whilst responses to questions on barriers and enablers mostly comprised a few succinct sentences, commonly using similar words and phrases. Following the analyses, survey respondents were provided with a summary of results gathered as a result of the survey and with reported examples of good practice in the implementation of NICE IP guidance.

## Results

### NHS hospital policies

**Fig. 1:**
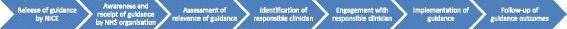
Implementation pathway following release of guidance

Consideration of the policies used by hospitals (identified in the supplementary policy documents review) allowed the typical pathway of implementation to be identified (Fig. 1). This presents potential steps taken by hospitals in the guidance implementation process and was used to structure the survey sent to NHS hospitals. This observed implementation pathway shown in Fig. 1 suggests a linear model is used. However, it may be that in practice a more pragmatic and less ordered approach is taken.

### Survey respondents

One hundred and thirty-five completed surveys (one response per hospital) were received from the 181 acute NHS hospitals within the UK, a response rate of 75 %. Five surveys were returned only partially completed and contained little or no qualitative data because respondents had mostly only completed tick box questions. Ninety percent of respondents (*n* = 122) provided their job title: they included Clinical Effectiveness, Governance and Safety Managers (*n* = 65), Clinical Audit Managers (*n* = 25), Board Directors and Medical Directors (*n* = 21).

The response rate varied between countries in the UK: Northern Ireland (80 %), England (75 %), Scotland (71 %) and Wales (57 %). Across the UK, the response rate was higher for teaching hospitals (79 %) than non-teaching hospitals (72 %). A standard chi-squared test was used to test if there was a difference in the number of responses received by teaching hospitals and non-teaching hospitals. The difference between these response rates was not significant (*χ*^2^ = 0.835, *p* = 0.36).

### Enablers and barriers to the implementation of IP guidance

One hundred and eighty-three comments were received in answer to open questions about the barriers and enablers to the implementation of IP guidance.

#### Enablers

**Fig. 2:**
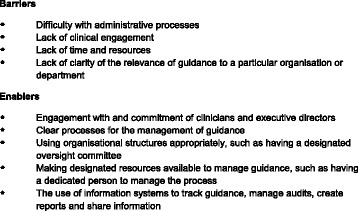
Key barriers and enablers of guidance implementation

The content analysis identified five broad themes of generic good practice, described as follows (see Fig. 2).

#### Engagement

Respondents frequently used terms such as ‘involvement’, ‘executive support’, ‘talk to clinicians’. Genuine engagement with clinicians and executive directors (clinical and non-clinical) and their commitment to the process were cited as a facilitator to guidance implementation. Senior clinician involvement on internal committees was described as helpful in solving problems with implementation of guidance, as well as the intervention of Clinical or Medical Directors, including those with specific responsibility for patient safety and quality. Respondents gave examples of senior clinician involvement on committees, including the chairing of a Steering Group and with monitoring the process at Directorate level, such as:

“[We] have executive support and consultants that are supportive with the process” [resp 9];

“Good feedback and engagement from the exec team” [resp 68].

Innovative ways of ensuring engagement were also cited, such as asking clinicians to undertake literature research to support new techniques or involving new clinicians and teams when they arrive at an organisation.

#### Clarity of processes

The use of effective and clear processes for the management of guidance was emphasised and the use of standardised, robust approaches allows clinicians to be aware of requirements and was said to boost their compliance. Organisations normally had designated individuals who coordinated the processes (these had already been observed in policies that were reviewed). The processes described awareness, dissemination, decision-making and monitoring and would normally be embedded in policies, implementation of which would be at a sub-organisational level, such as a clinical directorate.

Examples of this included:

“The process for approval of new procedures is robust and all clinicians are aware of this standardised approach to comply with the requirements” [resp 48];

“We have a robust process for the submission and approval of new interventional procedures” [resp 38];

“[We have a] newly launched flow chart for all staff to be aware of process timescales and interventions” [resp 20].

#### Committee-led implementation

Creating and utilising committees and hierarchies within hospitals, for example, discussing guidance issues on a monthly basis at a designated high-level committee, was cited as a facilitator to guidance implementation. A variety of committees were cited, such as Clinical Policy Groups, Clinical Guidance Groups, Trust Board Sub-Committee, Quality Panels and NICE Steering Group. This arrangement provides a forum for discussion relating to the implementation of guidance. Typical comments about this process were:

“We have the involvement of senior clinicians and board members in the allocation and monitoring NICE guidance implementation, and find it very useful that this process is carried out through a board subcommittee in ensuring a rapid response is provided” [resp 8];

“Each Clinical Directorate holds monthly Clinical Improvement Groups, so there is always a forum to enable discussion of implementation of NICE guidance” [resp 7].

#### Resources

In the survey responses, respondents reported that resources (financial and staffing) were critical to the implementation of any guidance that resources were scarce and resources for guidance implementation were in competition with other activities, such as audit. Assigning designated resources to managing guidance can, therefore, aid implementation. Examples included having a dedicated committee for guidance implementation and having a dedicated person to manage or coordinate the whole process of receiving new guidance.

#### Information systems

Use of information systems to track guidance, streamline reporting, manage audits, produce reports and share information can act as a facilitator. Several respondents commented that they used Microsoft® SharePoint as an electronic management system, CIRIS® to track guidance and manage audits and their action plans and Allocate Software® HealthAssure to manage reporting and monitoring. Respondents also told us of in-house databases, for example, with automatic reminders, to manage all guidance and action plans.

#### Local initiatives

Examples of local initiatives used to aid implementation of guidance were also provided, which were mostly about the sharing of information and the improvement of communication, such as a monthly newsletter of guidance related to clinical governance which included all new IP guidance published. Other methods discussed were presentation templates that ask key questions of clinicians wanting to implement a new procedure, guidance being recorded on a database which generates an email template to send to clinicians and advance warning of NICE IP guidance in development being sent to relevant specialists using web alerts.

#### Relationships with NICE

Respondents were also given the opportunity to provide suggestions to NICE about enhancing the relationships that hospitals have with NICE. Twenty-two (16 %) respondents commented on the positive value of regional representatives (of the NICE implementation team) or their generally positive relationship with NICE. Respondents felt that the value of the scarce resource of the NICE implementation team could be maximised through having local forums to discuss or share good practice, by providing examples of implementation at conferences and by the use of regional networks to broaden the scope of communication and to facilitate sharing of good practice.

### Barriers

#### Difficulties with processes

The most commonly cited barrier to implementation (in 37 % of comments; *n* = 67) was difficulty with administrative processes required to implement the guidance. These administrative difficulties included internal issues which related to either the complexity of the guidance or complexity of the hospital and those relating to issues with NICE processes, such as communication about when guidance is issued. Twenty-three percent (n=13 %) of comments related to problems with clinical engagement, because clinicians lacked the time and resources to become involved with driving the implementation of IP guidance. Twenty-one percent (*n* = 39) of comments mentioned limited finances and resources being a barrier to implementation. Seven percent of comments (*n* = 12) stated IP guidance is generally not relevant to their organisation, but nevertheless, scarce resources are still required to make an assessment on the relevance of guidance. A small proportion of comments (5 %, *n* = 9) stated that other types of NICE guidance take priority over IP guidance.

#### Targeting of guidance

Respondents commented on a need for improved targeting of IP guidance, through sending guidance only to those hospitals who would consider implementing the guidance. This, however, would likely be problematic with significant resources required to ascertain who may utilise the guidance. Many respondents commented that some IPs are not relevant to them, for example, because they do not have that specialty or they are not a teaching hospital that undertakes these procedures. Others reported a lack of clarity on the relevance of guidance to their hospital or department.

### Guidance dissemination processes and monitoring arrangements

The most frequent methods chosen by hospitals to be informed about new guidance were signing up to electronic reminders and/or actively reviewing relevant websites. Most survey respondents had a system in place to record the receipt or discovery of new guidelines. When the guidance had been received, decision-making about whether IP guidance was relevant in each hospital was undertaken at departmental level (40 % of respondents), by a designated individual (34 % of respondents) or by designated committees (25 % of respondents). Designated individuals included Medical Directors or Governance and Clinical Effectiveness Managers, whilst designated committees included Clinical Governance Committees or NICE Coordination Groups. These three levels of decision-making were also used to identify the clinician or manager responsible for implementing the new IP guidance.

**Fig. 3:**
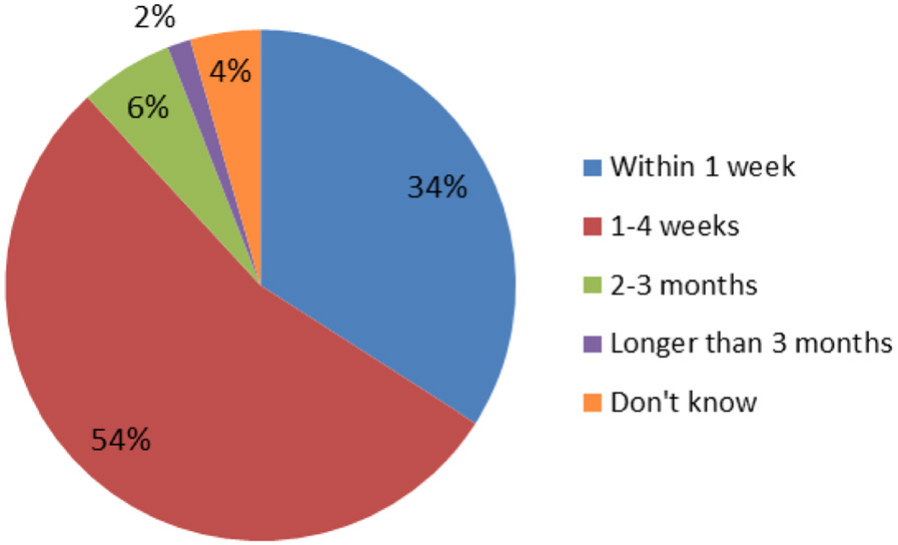
Lapse time between issue of IP guidance and contact with responsible person

**Fig. 4:**
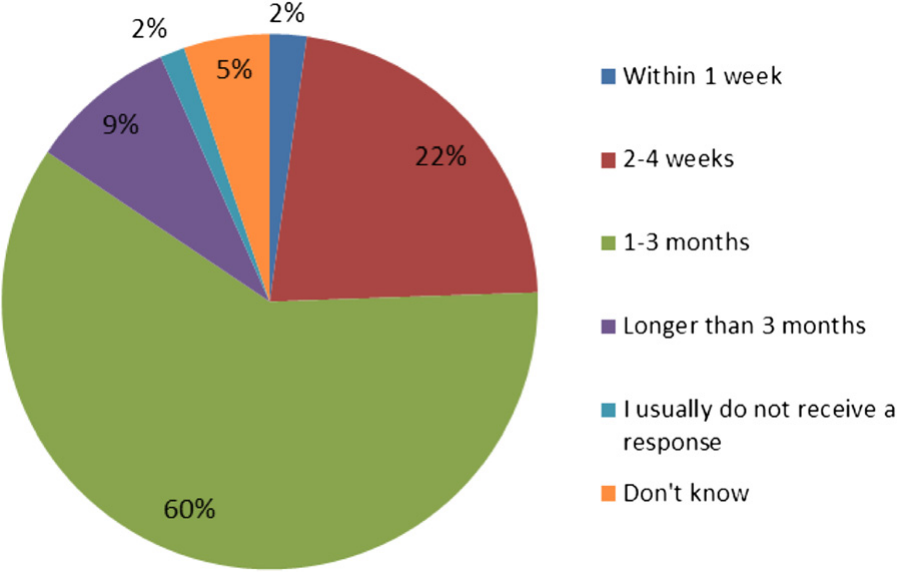
Lapse time between initial contact and locally judged satisfactory response

Respondents described robust processes for the dissemination of guidance, which was supported by publically available policies identified by the authors from websites of NHS hospitals. Forty-two respondents (32 %) provided examples of terms of reference and meeting notes supporting these processes. The average lapse time between the issuing of new IP guidance and disseminating the guidance through contact with the person responsible for implementing the guidance is shown in Fig. 3, whilst the average lapse time between initial contact and a locally judged satisfactory response is shown in Fig. 4. In situations where no response or an unsatisfactory response was received from the responsible clinician, robust processes for follow-up were in place. Follow-up processes included sending reminder emails and formal escalation to committees or the Medical Director.

Respondents described the processes that they used to confirm that IP guidance had been implemented satisfactorily. Both subjective and objective approaches were taken. In subjective approaches, such as by self-assessment, no evidence appeared to be collected or reviewed, whilst objective approaches, such as audit, required evidence and data to demonstrate compliance. Forty-five percent of comments (*n* = 57) described the use of audit through programmes within a specialty or the organisation’s annual audit plans. Clinical audit measures current practice against a defined standard, such as clinical guidance, to highlight where improvements may be made [16]. Although many respondents described the use of audit plans, there was often no comment on whether the plans were followed up or delivered. In cases where IP guidance only applied to a small number of patients, the lead clinician for the specialty concerned might be asked to provide short case summaries of individual patients.

## Discussion

This study identified the processes used by NHS acute hospitals to implement and monitor guidance. The main findings of this research identified key barriers and enablers to guidance implementation in NHS acute hospitals. These are discussed in more detail in the succeeding discussion. These findings can be used by both NHS acute hospitals and organisations like NICE, which produce guidance in order to make the process of implementing guidance easier.

Suggestions emerged for improving engagement with clinicians, as a vital element of the successful implementation of guidance. When clinicians have been involved in the production of guidance via an expert advisor or guidance consultee role for NICE, they and their colleagues are likely to be more engaged with implementing the guidance. More generally, identifying the lead clinician responsible for implementation of guidance *prior* to guidance publication was reported to aid in clinician engagement. It was not reported why prior notification aids engagement but, given the previous reports of time constraints, it is plausible that knowing about the guidance in advance allows for time resources to be better planned.

A number of actions were suggested through which organisations producing guidance might facilitate successful implementation. Improving the way in which the guidance is displayed online can allow users to identify new guidance more easily, whilst clear labelling and short summaries allow users to determine clinical specialities of relevance to guidance. Guidance producing organisations can also simplify the process for guidance users by making the issue of guidance routine and advertising guidance issue dates.

Another suggestion for organisations producing guidance, to enhance implementation, was to increase attention to shared learning and knowledge, to allow hospitals to learn from each other about areas of good practice. An example which was cited was the shared learning awards offered by NICE at its annual conference: organisations are invited to submit examples of good practice following implementation of guidance. This provides a useful platform for sharing knowledge, but it was suggested that more frequent and accessible forums may be useful. Responders to the survey encouraged measures to increase awareness of platforms such as the Shared Learning Database available via the NICE website which can assist users in sharing good practice [20]. This database is available for individuals to submit examples of practice with the aim of sharing learning among NHS and partner organisations. Where organisations are willing to share examples of good practice and guidance implementation, duplication of work can be avoided and experiences shared effectively.

The enablers identified by survey respondents that encourage successful behaviour change and, therefore, successful guidance implementation are echoed in the BCW proposed by Michie et al. [12]. Clinician engagement stimulates behaviour change in other staff. It is plausible to assume this may be a method of persuasion (as outlined in the BCW). Having clear processes and resources available enables the guidance to be implemented. Clear organisational structures, including committees, is defined in the BCW by environmental restructuring and through coercion. For example, when there is a non-response to new guidance, this is often escalated to committee level. Finally, sharing information is a form of education (as outlined in the BCW) which increases understanding within and between hospitals and hospital departments. Similar work by Sheldon et al., who looked at NICE guidance more generally, found that implementation of guidance was often left to an individual clinician with no tracking or audit of compliance and a lack of consultant engagement [3]. Compared with the findings of previous studies, the findings of the current study suggest that there has been some improvement in the processes associated with guidance implementation and monitoring. Many organisations reported robust processes for both the implementation and ongoing audit of guidance. Sheldon et al. concluded that organisations with good systems of tracking implementation were those most likely to adopt new guidance [3]. The current research suggests some improvement may have come about through more frequent use of technology to aid the process, with organisations using recognised software or developing in-house databases to help with all steps of the implementation programme, from tracking guidance to managing audits and producing reports.

However, there were examples within the current research of organisations experiencing the key problems highlighted by Sheldon et al. [3], for example, lack of clinician engagement or ‘consultant buy in’. When clinicians are fully engaged with the guidance, that is, they agree with the recommendations and the evidence to support those recommendations they are more likely to implement guidance. In addition, clinicians will implement guidance more successfully where time and resources exist to support the process.

The research by Sheldon et al. identified that inadequate funding for implementation of guidance recommendations was a barrier. Similarly, respondents in this research also highlighted scarce resources as a barrier to implementation [3]. A scarcity of staff time required to successfully implement guidance existed which included both administrative processes and clinician time. Respondents noted that even when a piece of guidance was not relevant to an organisation or department, a member of staff’s limited time was still necessary to reach this decision. Further, the supplementary review of hospitals' documents published online identified policies for implementing guidance in only 25 % of organisations, and many of these were outdated. The lack of policies and the fact that they are not being updated suggests that this is not an area of high priority for hospitals, probably because of competing demands. It is plausible, however, that more hospitals have up-to-date policies which we were unable to obtain from their publicly available information.

This study reports the results of a large-scale survey on the implementation of NICE IP guidance in acute hospitals. A high response rate (75 %) was achieved. However, there are a number of limitations to this study. First, the reasons that 25 % of hospitals did not respond are not known. Throughout this paper, it is assumed that this cohort of non-responders is not materially different to those that responded and that the sample of responders is a representative of all NHS acute hospitals. Although the non-responding hospitals were more likely to have non-teaching status, this difference was not statistically significant and the impact of this is unknown. Second, it was evident from a number of responses that the answers being provided related to guidance generally, rather than NICE IP guidance alone. Whilst this has allowed us to draw more generalisable conclusions, it calls into question the specific responses around IP guidance. Further, this study is limited by the method of data collection. As the survey was self-administered, responses may reflect ideal scenarios and policies rather than reality. Undertaking an audit of acute NHS hospitals would overcome this. Responders were able to remain anonymous in an attempt to generate reliable and honest data, but the extent to which this was successful is unknown. Although the survey focused on NICE IP guidance, the findings are relevant to the implementation of other types of guidance. Indeed, it was evident in the responses received that respondents were often discussing guidance implementation generally, in particular with respect to barriers and enablers for implementation.

## Conclusion

This survey of acute NHS hospitals was undertaken to inform about the understanding of the process of implementation of NICE IP guidance and to determine barriers and enablers to implementation within hospitals. Many hospitals have robust processes in place to deal with implementing guidance, but limited resources and the amount of guidance received by hospitals were reported as challenging. An important suggestion for improved implementation of guidance was the development of shared learning initiatives by organisations producing guidance and systems for open knowledge transfer between recipient organisations.

## Additional file

Additional file 1:
**Survey.** These questions are about the awareness of NICE Interventional Procedures Guidance and how it is received by the people’s organisation.

## Abbreviations

IP: interventional procedure
NICE: National Institute for Health and Care Excellence
