# Supplementary material for: Examining the implementation of NICE guidance: cross-sectional survey of the use of NICE interventional procedures guidance by NHS Trusts
Source: Implement Sci. 2015 Jun 30;10:93. doi: 10.1186/s13012-015-0283-4 (PMC4486420; doi:10.1186/s13012-015-0283-4)
Supplement: Additional file 1: — Survey. These questions are about the awareness of NICE Interventional Procedures Guidance and how it is received by the people’s organisation. [file 13012_2015_283_MOESM1_ESM.pdf]

Additional file 1

Section 1 These questions ask about the awareness of NICE Interventional Procedures Guidance and how it is received by your organisation

1. How are **<u>you</u>** (as the person responsible for the coordination and management of NICE Interventional Procedures Guidance) made aware of new NICE Interventional Procedures Guidance being issued? Please tick all that apply.

☐I review the NICE website daily
☐I review the NICE website weekly
☐I review the NICE website monthly
☐I review the NICE website infrequently on an ad hoc basis
☐I review the NICE Guidance Forward Planner
☐I have signed up for receipt of the electronic NICE News
☐I use the NICE phone app
☐Other (please state)

____

2. Does your organisation have a system in place for recording the receipt of NICE Interventional Procedures Guidance?

☐Yes
☐No
☐Don’t know

Section 2 These questions ask about the implementation processes for NICE Interventional Procedures Guidance in your organisation

3. How do you decide whether a NICE Interventional Procedures Guidance is relevant to your organisation?

____

____

4. (i) How do you decide who is the relevant clinician and/or manager responsible for the implementation of an individual guidance issued by the NICE Interventional Procedures programme?

____

____

(ii) How do you normally make initial contact with this clinician and/or manager to initiate implementation?

____

____

5. What is the <u>average</u> lapse time between you being aware that NICE Interventional Procedures Guidance has been issued on a procedure and making the initial contact with this responsible person?

☐Within 1 week
☐1-4 weeks
☐2-3 months
☐Longer than 3 months

6. How do you normally follow-up your initial communication if you are unhappy with their response or receive no response?

____

____

7. How do you confirm that the NICE Interventional Procedures Guidance has been implemented satisfactorily?

____

____

8. Can you provide an estimate of the <u>average</u> lapse time between you making the initial contact and receiving a satisfactory response from this responsible person?

☐Within 1 week
☐2-4 weeks
☐1- 3 months
☐Longer than 3 months
☐I usually do not receive a response

9. Does your organisation keep a register of all procedures carried out, which are covered by NICE Interventional Procedures Guidance requiring **special arrangements** and/or a **research only** protocol?

☐Yes
☐No
☐Don't know

Section 3 These questions ask about the processes for ensuring compliance with NICE Interventional Procedures Guidance

10. Please give the name of any committees in your organisation that consider implementation of NICE Interventional Procedures Guidance. Please can you also provide us with copies of their terms of reference, and if possible notes of one recent meetings?

____

____

11. What is the job title of the Executive Lead in your organisation accountable for the implementation of NICE Interventional Procedures Guidance?

____

12. **(i)** How often are reports containing information on the implementation of NICE Interventional Procedures Guidance, received by your organisation's Board of Directors?

☐Weekly
☐Monthly
☐Quarterly
☐Six-monthly
☐Annually
☐Ad hoc
☐None are received
☐Don't know

(ii) If these reports <u>are</u> received by your organisation's Board of Directors, please could you provide us with a copy of a recent report containing information on the implementation of NICE Interventional Procedures Guidance?

☐Attached
☐Not able to provide

(iii) If these reports are <u>not</u> received by your organisation's Board of Directors, please tell us how they receive information on the implementation of NICE Interventional Procedures Guidance?

____

____

____

Section 4 These questions ask about relationships with NICE, barriers and obstacles to the implementation of NICE Interventional Procedures Guidance, and examples of good practice

13. (i) Does your organisation find the clinical audit tools issued with a set of guidance helpful for implementing NICE Interventional Procedures Guidance?

☐Yes
☐No
☐I didn't know clinical audit tools were available
☐I have not used them
☐I don't know

(ii) If the clinical audit tools were not helpful, please tell us why.

____
____

14. Please suggest any tools or mechanisms that you think NICE could provide that would improve the process for the implementation of NICE Interventional Procedures Guidance.

____
____
____
____

15. Please suggest how relationships between your organisation and NICE could be developed in order to improve the implementation of NICE Interventional Procedures Guidance in your organisation.

____
____
____
____

16. Please give examples of any obstacles and barriers found by your organisation that hinder the implementation of NICE Interventional Procedures Guidance.

Examples of obstacles and barriers

17. Please give examples of good practice from your organisation that you would like to recommend and share with others which aid your implementation of NICE Interventional Procedures Guidance.

Examples of good practice and recommendations

18. Is there anything else you would like to tell us about any aspect of NICE Interventional Procedures Guidance?

____
____
____

Section 5 These questions ask for some background information which will assist us with the analysis of the survey data

19. What is the name of your organisation? (**Note:** your organisation name will **not** be used for anything other than to analyse data by size and type of organisation. Any identifiable information in this survey will remain confidential).

____

20. What is your job-title? (**Note:** your job title will be used for analysis purposes **only**. Any identifiable information in this survey will remain confidential).

____

THANK YOU VERY MUCH FOR YOUR HELP WITH THIS EVALUATION

Please can you give us your name and email address. (**Note**: this information is optional but will enable us to send to you examples of good practice from other organisations).

Name____
Email address____

NICE would also like to compile a contact list that they can use for notifying you of guidance issued and for sending to you other useful communications. Can you please tick the box below if you wish to be included on this contact list.

☐Yes, I have given permission to YHEC to provide NICE with my contact details (i.e. name and email address only)
